# Association of *GST* Genetic Polymorphisms with the Susceptibility to Hepatocellular Carcinoma (HCC) in Chinese Population Evaluated by an Updated Systematic Meta-Analysis

**DOI:** 10.1371/journal.pone.0057043

**Published:** 2013-02-20

**Authors:** Kui Liu, Lu Zhang, Xialu Lin, Liangliang Chen, Hongbo Shi, Ruth Magaye, Baobo Zou, Jinshun Zhao

**Affiliations:** 1 Department of Preventative Medicine and Zhejiang Provincial Key Laboratory of Pathological and Physiological Technology, School of Medicine, Ningbo University, Ningbo, Zhejiang Province, People’s Republic of China; 2 School of Health Management, Anhui Medical University, Hefei, Anhui Province, People’s Republic of China; 3 Yinzhou Peoples’ Hospital, Ningbo University, Ningbo, Zhejiang Province, People’s Republic of China; University of North Carolina School of Medicine, United States of America

## Abstract

**Background:**

Due to the possible involvement of Glutathione *S*-transferase *Mu-*1 (*GSTM1*) and Glutathione *S*-transferase *theta-1* (*GSTT1*) in the detoxification of environmental carcinogens, environmental toxins, and oxidative stress products, genetic polymorphisms of these two genes may play important roles in the susceptibility of human being to hepatocellular carcinoma. However, the existing research results are not conclusive.

**Methods:**

A systematic literature search using databases (PubMed, Scopus, Embase, Chinese Biomedical Database, Chinese National Knowledge Infrastructure, Wanfang Data, etc.) for the eligible studies meeting the inclusion criteria including case-control studies or cohort studies is evaluated using an updated systematic meta-analysis.

**Results:**

Significant increase in the risk of HCC in the Chinese population is found in *GSTM1* null genotype (OR = 1.47, 95% CI: 1.21 to 1.79, *P*<0.001) and *GSTT1* null genotype (OR = 1.38, 95% CI: 1.14 to 1.65, *P*<0.001). Analysis using the random-effects model found an increased risk of HCC in *GSTM1-GSTT1* dual null population (OR = 1.79, 95% CI: 1.26 to 2.53, *P*<0.001). In addition, subgroup analyses showed a significant increase in the association of *GST* genetic polymorphisms (*GSTM1*, *GSTT1*, and *GSTM1-GSTT1*) with HCC in southeast and central China mainland. However, available data collected by this study fail to show an association between *GST* genetic polymorphisms and HCC in people from the Taiwan region (for *GSTM1*: OR = 0.78, 95% CI: 0.60 to 1.01, *P* = 0.06; for *GSTT1*: OR = 0.94, 95% CI: 0.78 to 1.14, *P* = 0.546; for *GSTM1-GSTT1*: OR = 1.04, 95% CI: 0.81 to 1.32, *P* = 0.77). Sensitivity analysis and publication bias diagnostics confirmed the reliability and stability of this meta-analysis.

**Conclusions:**

Our results indicate that both *GSTM1* and *GSTT1* null genotypes are associated with an increased HCC risk in Chinese population. Peoples with dual null genotypes of *GSTM1-GSTT1* are more susceptible to developing HCC. In conclusion, *GST* genetic polymorphisms play vital roles in the development of HCC in the Chinese population.

## Introduction

Due to a high mortality, hepatocellular carcinoma (HCC) is one of the most serious health problems worldwide [1]–[2], which is consisted of approximately 80% of all primary tumors of liver [3]. Incidence rates in males and females are listed sixth and ninth as the most common cancers, respectively. Incidence rate of HCC has been increasing for several years while overall cancer incidence rate has been decreasing in recent years [4]–[5]. Environment and genetic factors are believed to be the pathogenesis of HCC [6]–[8]. Furthermore, previous studies indicated that racial and ethnic variations in the same geographic location could cause result bias in meta-analysis [9]–[11]. In Asia, people are at higher risk of developing HCC because of chronic infection with hepatitis B virus (HBV) [12]–[13]. In Europe, not only hepatitis C virus (HCV) and cirrhosis, but alcohol and tobacco smoking are also clearly able to accelerate HCC development [2]. Due to its substantial morbidity and mortality, HCC has been a hot research topic in China in recent years.

The Glutathione *S*-transferases (*GSTs*) family is an important phase II isoenzyme which can detoxify environmental carcinogens and toxins, oxidative stress products, and modulate the induction of other enzymes and proteins in the cell at the same time [14]–[16]. Enzymes of *GSTs* family are composed of many cytosolic, mitochondrial, and MAPEG proteins. Human *GSTs* can be divided into eight main classes including alpha, mu, pi, theta, sigma, kappa, omega and zeta [17]. *GSTM1* and *GSTT1* (encoding the Mu and Theta, respectively) both play important roles in human carcinogenesis. Epidemiologic investigations related to genetic association including case-control and cohort studies suggested the association between *GST* genetic polymorphisms and HCC risk. However, some of these studies with sparse data, unreasonable and highly underpowered designs, and differential in research methodology could all inevitably influence the robustness of their results. Meta-analysis can avoid these weaknesses by selecting all eligible studies and reducing random error. To identify the association of *GST* genetic polymorphisms with the susceptibility to hepatocellular carcinoma in the Chinese population, an updated systematic meta-analysis was performed in this study by using a full reference search (from January 1996 to October 2012) and a careful reinvestigation strategy.

## Methods

### 1. Literature and Research Strategy

A computerized literature search was carried out in Embase, PubMed, Scopus, Chinese Biomedical Database (CBM), CochraneLibrary, Chinese National Knowledge Infrastructure (CNKI), and Wanfang Data (the latest research was retrospected to October 2012) to collect articles with case-control or cohort studies related to the association of *GSTM1* and/or *GSTT1* polymorphisms with the susceptibility of HCC in China. Meanwhile, reference lists of the relevant articles were also collected. Search was performed through websites of http://www.baidu.com and http://scholar.google.cn to identify additional eligible studies. MeSH terms (“glutathione *S*-transferase” or “*GST*” or “*GSTM1*” or “*GSTT1*”) and (“hepatocellular carcinoma” or “liver cancer” or “HCC”) and (“China” or “Chinese” or “Taiwan”) were used in PubMed. These keyword retrieval strategies were also used in other databases. When there was more than one article published in a same case series, the latest and/or the comprehensive one would be adopted only. Eligible research articles not captured by above research strategies would be further searched by bibliographies.

### 2. Inclusion and Exclusion Criteria

Inclusion criteria are: (1) case-control and cohort studies, in which individuals or samples used for evaluation of the association between *GST* genetic variances and HCC risk included these owning either with a balance match or not; (2) in the Chinese population; (3) the articles provided raw data including odds ratio (OR) with 95% confidence interval (CI) and respective variance, or the relevant information could be calculated.

Exclusion criteria are: (1) raw data not available for retrieval; (2) multiple articles based on a same population and published by a same research team, only the latest and/or the largest population study was adopted, others would be excluded; (3) meeting abstract, case reports, editorials, review articles and other meta-analysis were exclusive.

### 3. Data Extraction and Synthesis

To decide inclusively or exclusively, articles were identified by two independent reviewers using a standardized data extraction form designed by our group. Data with discrepancies in identification were discussed. If consensus was not achieved, the decision was made by a third reviewer. Both title and abstract from all potential included articles were screened to identify their relevance. Additionally, if title and abstract were ambiguous, full articles were also investigated. The following information was collected from each study: first author, year of publication, geographical location, study time, pathologic diagnosis, source of control, characteristic of cases and controls, and genotype frequency of null *GSTM1*, *GSTT1* and null of both genotypes in cases and controls.

### 4. Statistical Analysis

(1) The pooled OR and 95% CI were determined by Z test with *P*<0.05 considered statistically significant; (2) Statistical heterogeneity among studies was assessed with the Q and I^2^ statistics [18]. The Q test and I^2^ were claimed to test the variation which was due to heterogeneity or by random error [19]. When *P* value of heterogeneity tests was no more than 0.1 (*P*≤0.1), we used random effects model. When *P* value of heterogeneity test was more than 0.1 (*P*>0.1), we used fixed effects model [20]; (3) Sensitivity analysis was also tested by removing one study at a time to calculate the overall homogeneity and effect size; (4) Publication bias was investigated with Beggar’s funnel plot, in which the standard error of log OR of each study was plotted against its OR [21]; (5) Publication bias was further assessed by the method of Egger’s linear regression test which could assess the relationship between effect size and variance differs between large and small studies [22]; (6) In this meta-analysis, subgroup analyses were used to better investigate possible reasons of between-study heterogeneity [23]. The subgroups are as following: geographical location (southeast and central China mainland, and Taiwan region), number of case (<100 *vs.* ≥100), source of control (population-based *vs.* hospital-based); (7) All analyses were performed using the software State version12.0 (StataCorp LP,College Station,Texas,USA), Review Manager 5.0 (Cochrane collaboration, http://www.cc-ims.net/RevMan/relnotes.htm). All the *P* values were two sided.

## Results

### 1. Study Selection and Study Characteristics

**Figure 1:**
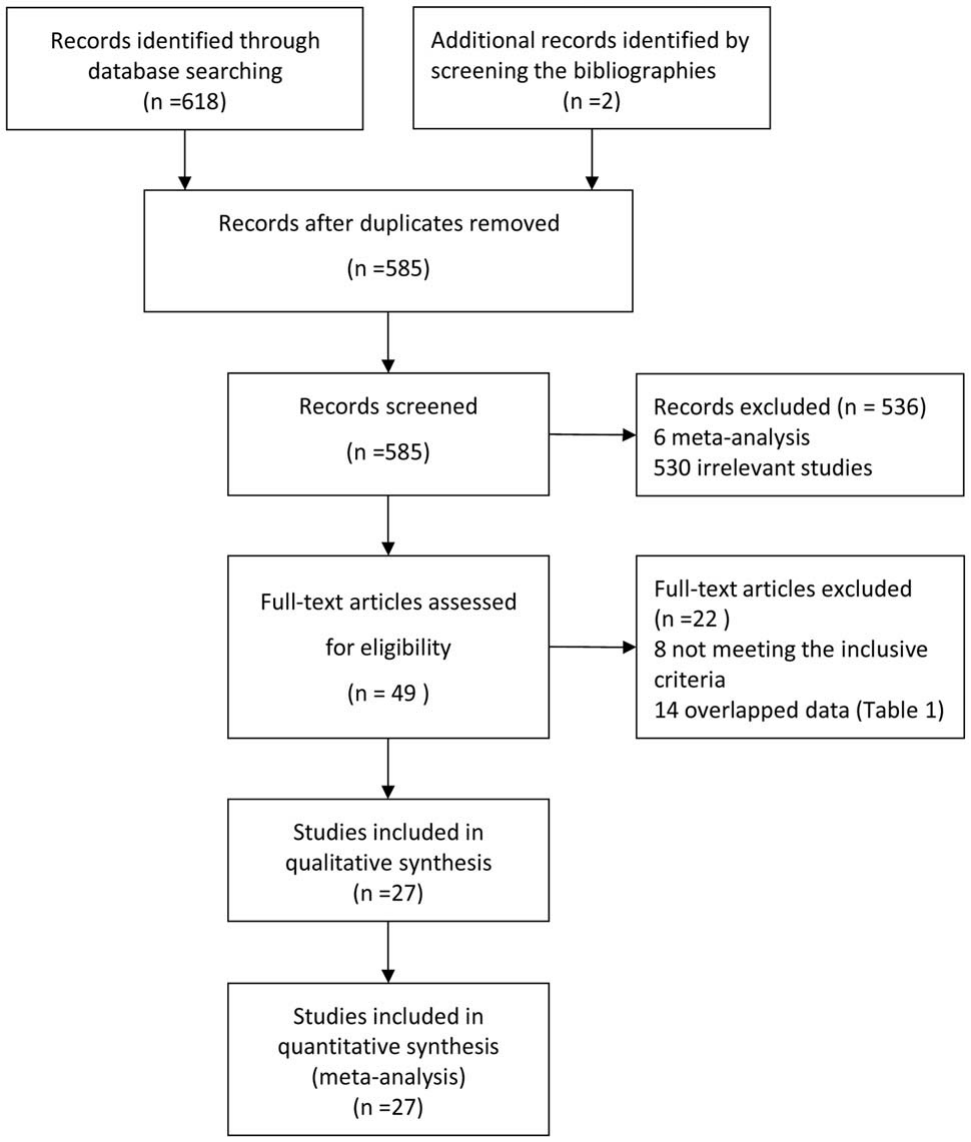
Flow chart of study selection.

We ultimately identified a total of 27 articles reporting the relationship between *GST* genetic polymorphisms and HCC risk by both Chinese and English database [24]–[50] (Figure 1). According to the inclusive and exclusive criteria, all articles were retrieved and carefully reviewed to assess the eligibility. The characteristics of the studies including 26 articles of *GSTM1* (3712 cases and 6024 controls), 21 articles of *GSTT1* (3378 cases and 5400 controls) and 12 articles of both *GSTM1* and *GSTT1* (1562 cases and 2537 controls) are shown in Table 1.

**Table 1:** Characteristics of the studies related with the effects of *GSTs* genetic polymorphisms and HCC risk.

No.	Study (ref.)	Region	Study time	Pathologic diagnosis	Source of controls	Case group	Control group	Null *GSTM1*/Group number			Null *GSTT1*/Group number			Dual Null/Group number		Overlapped (ref.)
								case	control		case	control		case	control	
*1	Hsieh LL 1996([24])	Taiwan	1990–1992	ALL	NA	46 male caseswith HBsAg (+)	88 male controls with HBsAg (+) matchedon age	25/46	47/88							
^2	Bian JC 1996([25])	Zhejiang,etc.	NA	ALL	Population	65 cases	106 healthy controls	44/65	50/106							
^3	Hu Y 1997([26])	Jiangsu	NA	NA	Population	45 cases	147 healthy controls without consanguineous relationship	37/45	75/147							
^4	Dong CH 1997([27])	Hebei, etc.	NA	NA	Hospital	110 cases	112 controls	62/110	50/112		63/110	42/112		36/110	20/112	[58]
*5	Dong CH 1998([28])	Jiangsu	1996	NA	Population	64 cases	64 healthy controls, matched on age and sex	29/56	24/58		33/56	23/58		21/56	9/58	
*6	Yu MW 1999([29])	Taiwan	1988–1996	PARTIAL	Population	84 cases (81HBsAg (+))	375 controls (153 HBsAg (−) and 222 HBsAg (+) ), matched on age etc	42/84	216/375		41/83	181/375				[59]–[61]
*7	Bian JC 2000([30])	Jiangsu, etc.	NA	ALL	Population	63 cases(47male)	88 healthy controls (67male), without consanguineous relationship	36/63	37/88		8/63	33/88		1/63	16/88	[62]
*8	Ma Y 2001([31])	Guangxi	NA	ALL	Population	120 cases	100 healthy controls without any tumors, matched on age and sex	71/120	52/100							
^9	Wu HL 2000([32])	Hunan	1997–1999	ALL	Population	54 cases(46 male)	136 healthy controls	38/54	62/136							
^10	Zhu WC 2001([33])	Guangdong	NA	ALL	Population	52 cases	100 healthy controls equally comparable in sex, age, birthplace and ethnicity	34/52	41/100							
*11	Sun CA 2001([34])	Taiwan	1991–1997	PARTIAL	Population	79 cases withHBsAg (+)	149 controls with HBsAg (+), matched on age, sex, residential township etc	26/69	77/128		30/67	77/128				[63]
^12	Liu CZ 2002([35])	Shanghai, etc.	NA	ALL	Population	84 cases	144 healthy controls, equally comparable in age and birthplace, but not in sex	56/84	69/144		34/84	36/144		23/84	19/144	
^13	Liu ZG 2003([36])	Guangxi	NA	ALL	Population	51 cases	53 healthy controls without any tumors,equally comparable in age and sex				28/51	18/53				
*14	Yu MW 2003([37])	Taiwan	1997–2001	PARTIAL	Population	577 caseswith HBsAg (+)	389 controls with HBsAg (+), matched on age and sex	322/577	231/389		298/577	199/389		171/577	116/389	[51]
*15	McGlynn KA 2003([38])	Jiangsu	1992–1993	PARTIAL	Population	231 cases (73%HBsAg (+))	256 controls matched on age, sex and township of residence	OR (95% CI) = 0.83 (0.57, 1.21)§			OR(95% CI) = 0.88 (0.59, 1.31)§					
^16	Li SP 2004([39])	Jiangsu	1998–2002	NA	Population	207 cases	207 healthy controls, matched on sex, age and residence	122/207		118/207	108/207		97/207			
^17	He SJ 2004([40])	Guangxi	2001–2002	ALL	Population	105 HCC cases	151 healthy controls equally comparable in age, sex, ethnicity	68/105		77/151	43/105		50/151	30/105	31/151	[64], [65], [66]
^18	Guo HY 2005([41])	Henan	1999–2002	PARTIAL	Population	95 HCC cases	103 healthy controls equallycomparable in age, sex, residence	67/95		52/103	58/95		45/103	39/95	21/103	
^19	Ma DL 2005([42])	Guangxi	2003–2004	ALL	Population	62 cases withHBsAg (+)	73 controls with HBsAg (+), without any tumor,equally comparable in age and sex	37/62		29/73	35/62		21/73			
*20	Long XD 2005([43])	Guangxi	2002–2003	ALL	Hospital	140 cases	536 controls without any tumor,equally comparable in sex, age, ethnicity	92/140		254/536	82/140		234/536	60/140	127/536	[67]
*21	Deng ZL 2005([44])	Guangxi	1998–2002	ALL	Population	181 cases	360 controls without any tumor	117/181		172/360	108/181		154/360	38.2%	18.5%	[68], [69]
*22	Long XD 2006([45])	Guangxi	2004–2005	ALL	Population	257 cases	649 controls without clinical evidence of liver disease,matched on age, sex, ethnicityand HBV infection	179/257		312/649	146/257		297/649			
^23	Yang ZG 2009([46])	Guangxi	2002–2008	ALL	Population	100 cases	60 healthy controls withouthepatitis virus infection, tumorsand AFP negative, equally comparable in age and sex	59/100		41/60	33/100		11/60	22/100	2/60	
*24	Kao CC 2010([47])	Taiwan	2006–2008	ALL	Population	102 cases	386 healthy controls, matched onethnicity, sex and residential area	54/102		211/386	51/102		200/386	31/102	104/386	
*25	Wei YP 2012([48])	Guangxi	NA	ALL	Hospital	181 cases (78.5%HBsAg (+))	641 controls (9.8%HBsAg (+))without cancer disease, matched on age and sex	118/181		305/641	104/181		276/641			[70]
^26	Tang YT 2012([49])	Guangxi	2008–2010	ALL	Population	150 malecases	150 male healthy controls, equallycomparable in age	76/150		77/150	63/150		68/150	30/150	32/150	
*27	Ling CG 2012([50])	NA	2005–2007	ALL	Population	476 cases (54.7%HBsAg (+), 13.4%Anti-HCV (+))	481 controls (43.6%HBsAg (+), 2.5%Anti-HCV (+)), withoutmalignancy diseases etc., equally comparable in age and sex	244/476		211/481	120/476		94/481			

ALL: HCC cases were confirmed by pathologic diagnosis; PARTIAL: part of HCC cases were confirmed by pathologic diagnosis; NA: relative data were not available in original studies;

* Articles published in English;

^ Articles published in Chinese.

§ McGlynn et al. did not show genotype frequency of cases and controls, but presented OR with 95% CI;

Southeast regions in China mainland include Hebei, Shanghai, Jiangsu, Zhejiang, Anhui, Jiangxi, and Guangxi. Central regions in China mainland include Hunan and Henan.

### 2. Meta-analysis Results

#### 2.1. *GSTM1* null genotype with HCC risk

**Figure 2:**
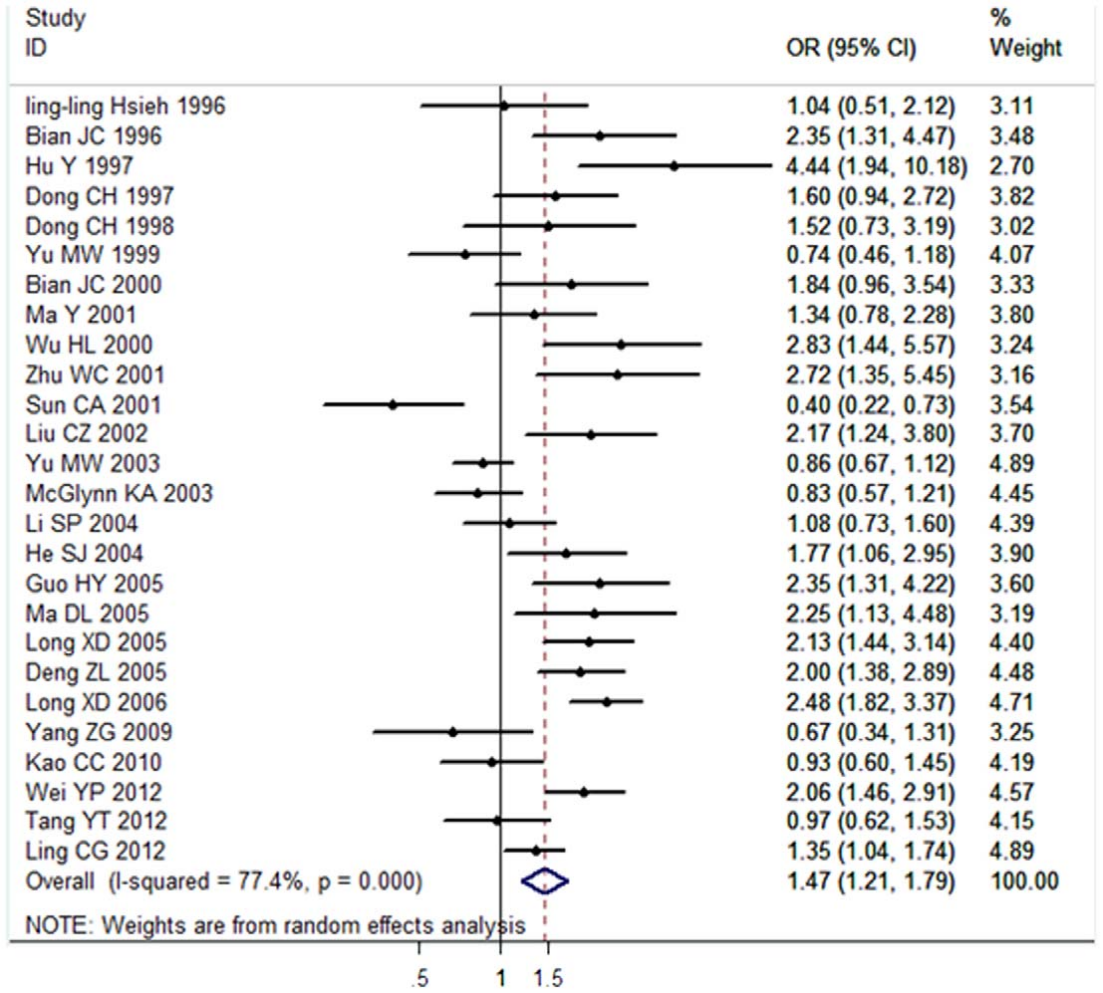
Association between *GSTM1* null genotype and HCC risk analyzed by forest plot of meta-analysis. The forest plots of pooled OR with 95% CI (Null genotype *vs.* Present genotype; OR = 1.47, 95% CI: 1.21 to 1.79; Random-effects model, *P*<0.001).

26 articles [24–35, and 37–50] including 3712 cases and 6024 controls were investigated in this study to evaluate the association between *GSTM1* null genotype and HCC susceptibility. 12 articles were published in Chinese and 14 articles in English. Results obtained from a random-effects model showed a significant association between the *GSTM1* null genotype and HCC risk in the Chinese population (OR = 1.47, 95% CI: 1.21 to 1.79, *P*<0.001). The forest plot was showed in Figure 2.

#### 2.2. *GSTT1* null genotype with HCC risk

**Figure 3:**
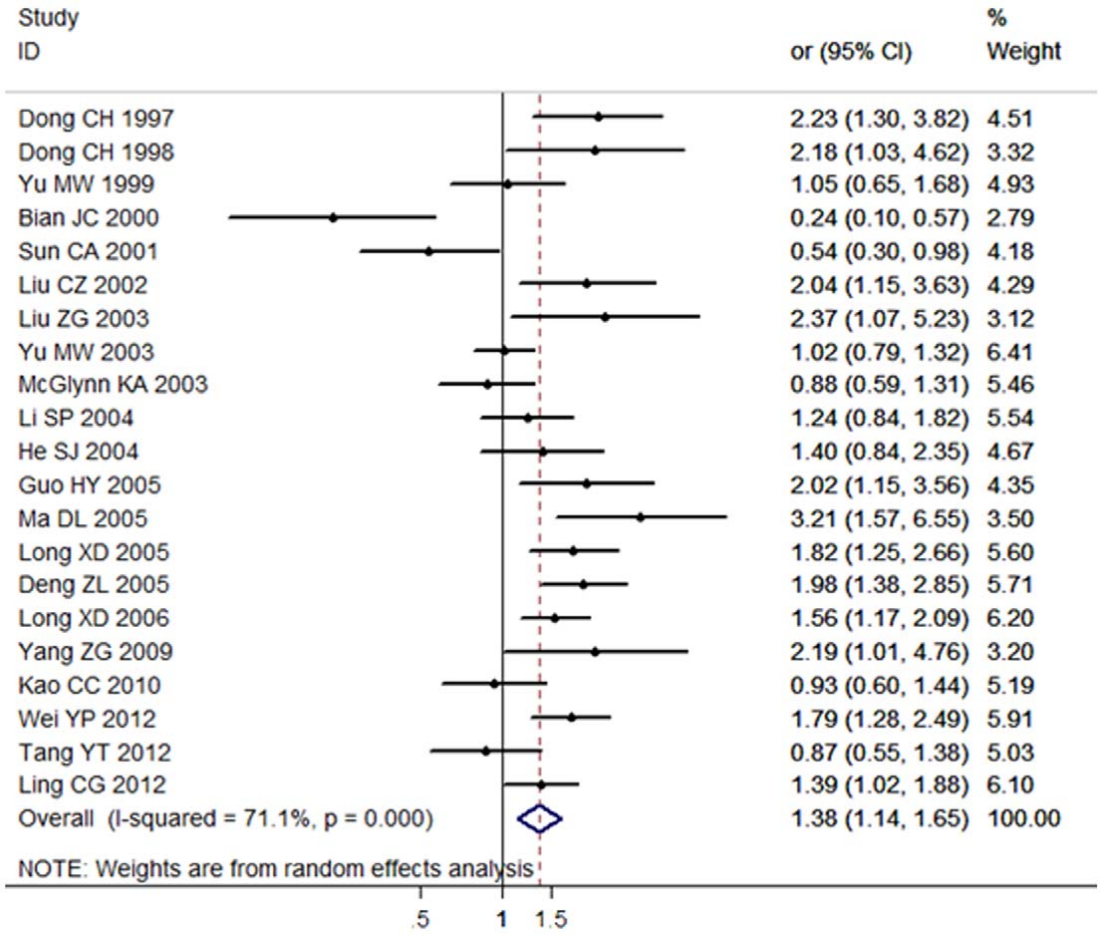
Association between *GSTT1* null genotype and HCC risk analyzed by forest plot of meta-analysis. The forest plots of pooled OR with 95% CI (Null genotype *vs.* Present genotype; OR = 1.38, 95% CI: 1.14 to 1.65; Random-effects model, *P*<0.001).

21 articles including 3378 cases and 5400 controls were used for the investigation of the association between *GSTT1* null genotype and HCC susceptibility. 9 articles were published in Chinese and 12 articles were published in English. Results showed that the *GSTM1* null genotype was significantly associated with HCC risk demonstrated by random-effects model in the Chinese population (OR = 1.38, 95% CI: 1.14 to 1.65, *P*<0.001). The forest plot was shown in Figure 3.

#### 2.3. Dual-null genotype of *GSTM1-GSTT1* with HCC risk

**Figure 4:**
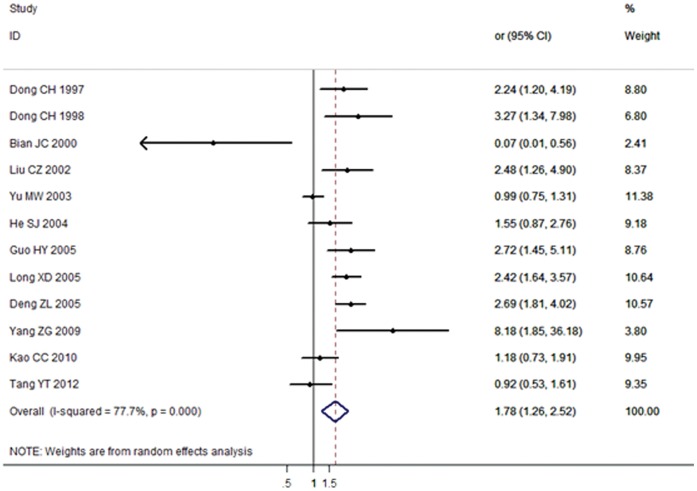
Association between *GSTM1-GSTT1* dual-null genotype and HCC risk analyzed by forest plot of meta-analysis. The forest plots of pooled OR with 95% CI (Dual-null genotype *vs.* Present genotype; OR = 1.79, 95% CI: 1.26 to 2.53; Random-effects model, *P*<0.001).

12 articles (6 articles in Chinese and 6 articles in English) including 1763 cases and 2537 controls were used to evaluate the relationship between *GSTM1-GSTT1* null genotype and HCC susceptibility. Results indicated that dual-null genotype of *GSTM1-GSTT1* also had a significant association with HCC risk in the Chinese population (OR = 1.79, 95% CI: 1.26 to 2.53, *P*<0.001). The forest plot was shown in Figure 4.

### 3. Subgroup Analysis

The substantial between-study heterogeneity of the three above analyses were observed (*P* values for *GSTM1*, *GSTT1*, and the interaction of *GSTM1-GSTT1* were all less than 0.001, I^2^ values were 77.4%, 71.1%, and 77.7%, respectively). In this meta-analysis, subgroup analyses contained geographical location (southeast regions in China mainland, central regions in China mainland, and Taiwan region), case number (<100 *vs.* ≥100), source of control (population-based *vs.* hospital-based). The between-study heterogeneity showed that the major source of heterogeneity came from China mainland population. Association between *GST* genetic polymorphisms and HCC risk increase was significant in subgroup analyses of both southeast and central regions in China mainland population, but no significant in Taiwan population. Other subgroup analyses results were shown in Table 2, Table 3, and Table 4.

**Table 2:** Subgroup analysis of the association between *GSTM1* null genotype and HCC risk.

Polymorphism	Null *vs*. Present	No. of studies (cases/controls)	Odds ratio		M	Heterogeneity		*P_E_*
			OR [95% CI]	*POR*		I^2^ (%)	*PH*	
*GSTM1*	All studies	26(3712/6024)	1.47[1.21,1.79]	<0.001	R	77.4%	<0.001	0.367
	subgroup analyses by geographical location							
	Southeast regions in mainland China	18(2209/3938)	1.69[1.38,2.07]	<0.001	R	67.0%	<0.001	0.805
	Central regions in mainland China	2(149/239)	2.55[1.64,3.97]	<0.001	F	0.0%	0.680	@
	Taiwan province	5(878/1366)	0.78[0.60,1.01]	0.06	F	38.1%	0.164	0.555
	subgroup analyses by number of case							
	<100	12(775/1546)	1.59[1.33,1.90]	<0.001	R	77.8%	<0.001	0.031
	≥100	14(2937/4478)	1.36[1.23,1.50]	<0.001	R	78.4%	<0.001	0.859
	subgroup analyses by source of control							
	population-based	21(3133/4261)	1.47[1.17,1.84]	<0.001	R	79.4%	<0.001	0.238
	hospital-based	4(533/1675)	1.62[1.11,2.37]	0.012	R	69.1%	0.021	0.472

M: model of meta-analysis; R: random-effects model; F: fixed-effects model. *P_H_*: *P* value of heterogeneity test. *P_E_*: *P* value of Egger’s test. *P_OR_*: *P*<0.001 replace *P* = 0.000 and *P* less than 0.001. @: *P* values could not be calculated.

**Table 3:** Subgroup analysis of the association between *GSTT1* null genotype and HCC risk.

Polymorphism	Null *vs.* Present	No. of studies (cases/controls)	Odds ratio		M	Heterogeneity		*P_E_*
			OR [95% CI]	*POR*		I^2^ (%)	*PH*	
*GSTT1*	All studies	21(3378/5400)	1.38[1.14,1.65]	<0.001	R	71.1%	<0.001	0.795
	subgroup analyses by geographical location							
	Southeast regions in mainland China	16(2454/4019)	1.51[1.35,1.69]	<0.001	R	67.1%	<0.001	0.952
	Central regions in mainland China	1(95/103)	2.02[1.15,3.56]	0.020	F	@	@	@
	Taiwan province	4(829/1278)	0.94[0.78,1.14]	0.546	F	24.4%	0.265	0.315
	subgroup analyses by number of case							
	<100	8(561/1022)	1.34[0.78,2.28]	0.258	R	81.8%	<0.001	0.961
	≥100	13(2817/4378)	1.38[1.16,1.64]	0.002	R	61.0%	<0.001	0.560
	subgroup analyses by source of control							
	population-based	17(2845/3725)	1.32[1.06,1.64]	<0.001	R	72.1%	<0.001	0.746
	hospital-based	4(533/1675)	1.60[1.14,2.26]	0.007	R	63.6%	0.041	0.929

M: model of meta-analysis; R: random-effects model; F: fixed-effects model. *P_H_: P* value of heterogeneity test. *P_E_*: *P v*alue of Egger’s test. *P_OR_*: *P*<0.001 replace the *P* = 0.000 and the *P* less than 0.001. @: *P* values could not be calculated.

**Table 4:** Subgroup analysis of the association between *GSTM1-GSTT1* null genotype and HCC risk.

Polymorphism	Null vs. Present	No. of studies (cases/controls)		Odds ratio			M	Heterogeneity		*P_E_*
				OR [95% CI]		*POR*		I^2^ (%)	*PH*	
*GSTM1-GSTT1*	All studies	12(1763/2537)		1.78[1.26,2.52]		<0.001	R	77.7%	<0.001	0.535
	subgroup analyses by geographical location									
	Southeast regions in mainland China	9(989/1659)		1.98[1.32,2.95]	<0.001		R	70.3%	<0.001	0.497
	Central regions in mainland China	1(95/103)		2.72[1.45,5.11]	0.002		F	@	@	@
	Taiwan province	2(679/775)		1.04[0.81,1.32]	0.770		F	0.0%	0.536	@
	subgroup analyses by number of case									
	<100		4(298/393)	1.73[0.70,4.28]	0.235		R	75.9%	0.001	0.115
	≥100		8(1465/2144)	1.70[1.17,2.48]	0.006		R	78.8%	0.001	0.263
	subgroup analyses by source of control									
	population-based		9(1411/1503)	1.75[1.09,2.80]	0.020		R	81.0%	0.001	0.531
	hospital-based		3(352/1034)	1.86[1.16,2.97]	0.010		R	63.8%	0.063	0.856

M: model of meta-analysis; R: random-effects model; F: fixed-effects model. *P_H_*: *P* value of heterogeneity test. *P_E_*: *P* value of Egger’s test. *P_OR_*: *P*<0.001 replace the *P* = 0.000 and the *P* less than 0.001. @: *P* values could not be calculated.

### 4. Sensitivity and Heterogeneity Analysis

**Figure 5:**
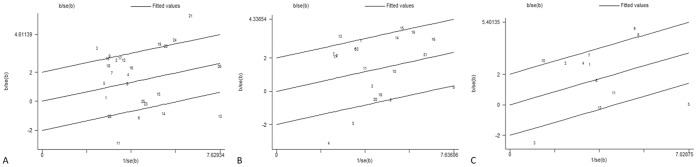
Galbraith plot of association between *GST* polymorphisms and HCC risk. Each figure represents a unique article in this meta-analysis. The figures outside the three lines are spotted as the outliers and the possible sources of heterogeneity in the analysis pooled of total available studies. (A) Galbraith plot identifies the outliers from 26 studies about *GSTM1* polymorphisms and HCC risk. (B) Galbraith plot identifies the outliers from 21 studies about *GSTT1* polymorphisms and HCC risk. (C) Galbraith plot identifies the outliers from 12 studies about *GSTM1-GSTT1* polymorphisms and HCC risk.

Sensitivity analysis was performed by sequential excluding one article each time. The significance of all ORs was not changed. We used Galbraith plot to omit some possible major sources of heterogeneous articles. The results were showed in Figure 5. In Figure 5A, we found more than 6 articles (No. 3, 6, 11, 13, 14, and 21) spotted by Galbraith plot. However, it might cause some biases by excluding those articles as the sources of heterogeneity. So we didn’t reduce the obvious between-study heterogeneity in the analyses on the *GSTM1* polymorphisms. In Figure 5B, 3 articles (No. 4, 5 and 13) were obviously spotted as the outliers and the possible sources of heterogeneity in the analysis pooled of total available studies, but another 3 articles (No. 8, 9 and 15) the outliers were not reduced because it could cause some biases. After adjustment, the association between *GSTT1* polymorphisms and HCC risk was increased (OR = 1.45, 95% CI: 1.24 to 1.69, *P*<0.001, random-effects model). Galbraith plots (Figure 5C) spotted 5 articles (No. 3, 5, 8, 9 and 10) as the possible sources of heterogeneity, but only 3 articles (No. 3, 5 and 9) were omitted for the obvious between-study heterogeneity in the analyses on the *GSTM1-GSTT1* polymorphisms. The adjusted OR and 95% CI between *GSTM1*/*GSTM1-GSTT1* polymorphisms and HCC risk was significantly increased although heterogeneity (I^2^ *_GSTT1_* = 56.1%, *P_GSTT1_* = 0.002; I^2^ *_GSTM1-GSTT1_* = 59.0%, *P_GSTM1-GSTT1_* = 0.012) still existed. These results were shown in Table 5 and Table 6.

**Table 5:** Subgroup analysis of $the adjusted association between *GSTT1* null genotype and HCC risk.

Polymorphism	Null *VS.* Present	No. of studies(cases/controls)	Odds ratio		M	Heterogeneity		*P_E_*
			OR [95% CI]	*POR*		I^2^ (%)	*PH*	
*GSTT1*	All studies	18(3186/5111)	1.45[1.24,1.69]	<0.001	R	56.1%	0.002	0.142

M: model of meta-analysis; R: random-effects model; F: fixed-effects model. *P_H_*: *P* value of heterogeneity test. *P_E_*: *P* value of Egger’ test. *P_OR_*: *P*<0.001 replace the *P* = 0.000 and the *P* less than 0.001.

$ adjusted association (after omitting 3 articles [30], [34], [42]).

**Table 6:** Subgroup analysis of ^$^the adjusted association between *GSTM1-GSTT1* null genotype and HCC risk.

Polymorphism	Null *vs.* Present	No. of studies (cases/controls)	Odds ratio		M	Heterogeneity		*P_E_*
			OR [95% CI]	*POR*		I^2^ (%)	*PH*	
*GSTM1-GSTT1*	All studies	9(942/1674)	1.98[1.43, 2.74]	<0.001	R	59.0%	0.012	0.236

M: model of meta-analysis; R: random-effects model; F: fixed-effects model. *P_H_*: *P* value of heterogeneity test. *P_E_*: *P* value of Egger’s test. *P_OR_*: *P*<0.001 replace the *P* = 0.000 and the *P* less than 0.001. ^$^: adjusted association (after omitting 3 articles [30], [37], [44]).

### 5. Potential Publication Bias

**Figure 6:**
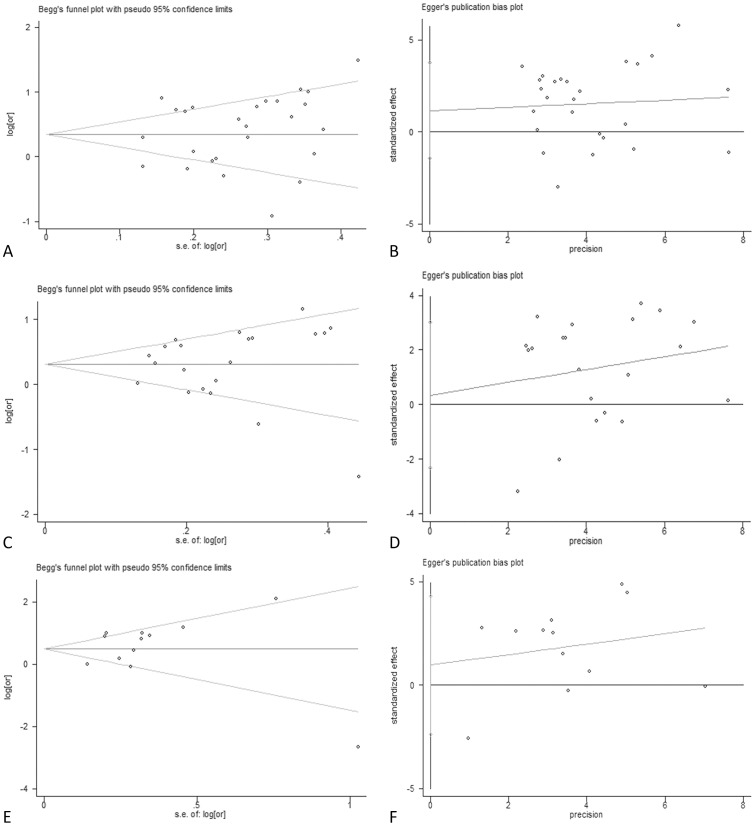
Beggar’s test and Egger’s test of *GST* polymorphisms and HCC risk. Beggar’s funnel plot is used to detect potential publication bias in which a symmetric funnel shape means no publication bias. Egger’s linear regression test is used to quantify the potential presence of publication bias. Both Beggar’s test and Egger’s test show that no publication bias has been found from 26 inclusive studies about the association between *GSTM1* polymorphisms and HCC risk (A and B), 21 inclusive studies about the association between *GSTT1* polymorphisms and HCC risk (C and D), and 12 inclusive studies about the association between dual-null genotype of *GSTM1-GSTT1* and HCC risk polymorphisms and HCC risk (E and F).

Beggar’s funnel plots and Egger’s publication bias plots were used to assess the potential publication bias for *GSTM1*, *GSTT1*, and dual-null genotype of *GSTM1-GSTT1* (Figure 6). No publication bias was detected by Egger’s test (*P_E_* = 0.367 for *GSTM1*, *P_E_* = 0.795 for *GSTT1* and *P_E_* = 0.64 for dual-null genotype of *GSTM1-GSTT1*).

## Discussion

The association between *GST* genetic polymorphisms and HCC risk are inconsistent according to the present research results. This may be caused by several reasons. Improper matching or insufficient case and control numbers used in the studies are all possible reasons. One meta-analysis [10] published in 2009 with the association between *GST* genetic polymorphisms and HCC risk didn’t cover all conclusive articles published in Chinese and English databases. In this meta-analysis paper, overlapped data was found in two adopted studies [37], [51] (two different articles with different case and control numbers written by the same research group). Another two adopted studies [52], [53] in this meta-analysis didn’t match properly for the cases (HBV carried) and controls (HBV negative). The other meta-analysis [11] published in 2012 about Asian population included a study [^38^] with unclear case and control numbers. In addition, some more studies [47]–[50] related with the association between *GST* genetic polymorphisms and HCC risk have emerged since these two meta-analysis papers were published.

To evaluate the association of *GST* genetic polymorphisms and susceptibility to HCC in the Chinese population, we performed an updated systematic meta-analysis. In this study, 27 articles (3781 patients and 6104 controls) were selected from Chinese and English databases. 26 studies (3712 cases and 6024 controls) out of the 27 articles were used for investigation of the relationship between *GSTM1* null genotype and HCC susceptibility. 21 studies (3378 cases and 5400 controls) out of the 27 articles were used to evaluate the relationship between *GSTT1* null genotype and HCC susceptibility. 12 studies (1763 cases and 2537 controls) were applied for evaluation for the *GSTM1-GSTT1* gene. Random-effects model of meta-analysis shows significant associations of polymorphisms of *GSTM1* null gene (OR = 1.47, 95% CI: 1.21 to 1.79, *P*<0.001), *GSTT1* null gene (OR = 1.38, 95% CI: 1.14 to 1.65, *P*<0.001), and *GSTM1-GSTT1* dual null gene (OR = 1.79, 95% CI: 1.26 to 2.53, *P*<0.001), respectively, with HCC risk in the Chinese population. Subgroup analyses on *GSTM1* null gene indicate that geographical location (China mainland, but not in Taiwan region), case numbers and source of controls are significantly associated with HCC risk. Results of subgroup analyses on *GSTT1* null gene and *GSTM1-GSTT1* dual null gene indicate that geographical location (China mainland, but not in Taiwan region), case numbers (≥100, but not <100) and source of controls are also significantly associated with HCC risk. Reasons for inconsistent in conclusions between China mainland and Taiwan region may be caused by environmental factors. Moreover, limited investigative numbers of the case-control/followed up studies from Taiwan region may result in difficulty for getting stable risk estimation, though these investigations own low between-study heterogeneity. In addition, studies with case number less than 100 may have effects on drawing a proper estimation for the association between *GST* genetic polymorphisms and HCC risk. Therefore, further well design case-control/followed-up studies, especially with a larger case number, are necessary to provide better evidences for the evaluation. Heterogeneity analysis is a key part of meta-analysis. Q statistic test (Cochran’s Q statistic) and I^2^ statistic test are commonly used to test and quantify the between-study heterogeneity. The major source of heterogeneity in the China mainland population detected in the subgroup analysis might come from the environmental difference which could affect their sensitivity to particular genomic variants. In this meta-analysis, Galbraith plot was performed for identifying the articles with possible heterogeneity. However, in the analyses on the *GSTM1* polymorphism and HCC risk, we kept several articles with obvious between-study heterogeneity because too many articles omitting could cause some biases. For the association of *GSTT1* null gene and HCC risk, we deleted 3 articles [30], [34], [42] which were obviously spotted as the outliers with major source of between-heterogeneity, and same procedures were done for *GSTM1-GSTT1* gene (3 article deletion [30], [37], [44]). Regretfully, the between-heterogeneity didn’t decrease significantly even if the adjustment was done in both *GSTT1* and *GSTM1-GSTT1* genetic polymorphisms (I^2^ *_GSTT1_* = 56.1%, *P_GSTT1_* = 0.002; I^2^ *_GSTM1-GSTT1_* = 59.0%, *P_GSTM1-GSTT1_* = 0.012). Therefore, we applied the random-effects model to evaluate the pooled OR for *GSTT1* and *GSTM1-GSTT1* genes, respectively. After the above adjustments, the associations were increased between *GSTT1* and *GSTM1-GSTT1* polymorphisms and HCC risk (OR*_GSTT1_* = 1.45, 95% CI: 1.24 to 1.69; OR*_GSTM1-GSTT1_* = 1.98, 95% CI: 1.43 to 2.74). In this study, Beggar’s funnel plots and Egger’s linear regression test were applied to assess the potential publication bias. No publication bias was detected (*P_E_* = 0.367 for *GSTM1*, *P_E_* = 0.795 for *GSTT1* and *P_E_* = 0.64 for dual-null genotype of *GSTM1-GSTT1,* Egger’s linear regression test).

Research evidences suggest that *GST* genetic polymorphisms are associated with the susceptibility to several carcinomas. Takahiko Katoh *et al.* [54] showed the *GSTM1* null genotype might be associated with susceptibility to gastric adenocarcinoma and distal colorectal adenocarcinoma in Japanese population. Wang J *et al*. [55] found that the combination of *GSTM1* null and *GSTP1* Val was significantly associated with an elevated lung adenocarcinoma risk (OR = 2.4, 95% CI: 1.1 to 5.1). Helzlsouer K J *et al*. [56] considered that genetic variability in members of the *GST* gene family might be associated with an increased susceptibility to breast cancer (OR = 3.77, 95% CI: 1.10 to 12.88). Compared to the control group value of 41.8%, Zhong S *et al*. [57] found a significant excess of 56.1% *GSTM1* gene null individuals in colorectal cancer group. Our meta-analysis results demonstrate that there is an association between *GST* genetic polymorphisms and susceptibility to HCC in the Chinese population. Thus, further epidemiological and molecular biological studies are necessary to clarify the role of *GST* genetic polymorphisms in HCC and other carcinomas.

Nevertheless, there were several limitations to this meta-analysis. (1) Observational studies were susceptible to various biases such as selection bias. Due to some studies without clear explanation for the pathologic diagnostic results of all/part subjects (Table 1), therefore, some selection bias might be unavoidable. (2) In some studies, participants in control groups stemmed from hospital-based population might not fully represent the population-based controls, which could distort the results (Table 1). (3) The conclusions draw from subgroup analysis might be limited due to a low statistic power from the small sample size. (4) Each study had its own inclusive criteria. For example, some studies selected from HbsAg positive population, while others selected the common people or healthy population. Due to these reasons, some bias might bring influence on the results. (5) Not only genetic polymorphisms but other factors such as alcohol consumption, AFB1 status, and chronic infection of HBV/HCV might also play vital roles in the development of HCC. Owning to the lack of sufficient data, gene-environment interactive functions were not evaluated in this meta-analysis, which might also have an influence on the precision of the conclusion.

In summary, our results suggest *GST* genetic polymorphisms are associated with the increased risk of HCC in the Chinese population. To further evaluate gene-to-gene and gene-to-environment combined effects on *GST* genetic polymorphisms and HCC,both large scale multicenter epidemiological studies in total population and/or selected population with different environmental background are urgently needed.
