# Correction: Association of *GST* Genetic Polymorphisms with the Susceptibility to Hepatocellular Carcinoma (HCC) in Chinese Population Evaluated by an Updated Systematic Meta-Analysis

**DOI:** 10.1371/annotation/13ee8778-996e-4649-af2e-4390378c2493

**Published:** 2014-01-29

**Authors:** Kui Liu, Lu Zhang, Xialu Lin, Liangliang Chen, Hongbo Shi, Ruth Magaye, Baobo Zou, Jinshun Zhao

The method employed for the evaluation of publication bias in this meta-analysis is incorrectly referred to as "Beggar's funnel plot" in the article (Statistical Analysis section within the Methods, Potential Publication Bias section within the Results, legend of Figure 6, and Discussion section). The method should have been cited as Begg's funnel plot. The authors apologize for this error.

